# Incidence of Neonatal Abstinence Syndrome Epidemic and Associated Predictors in Nevada: A Statewide Audit, 2016–2018

**DOI:** 10.3390/ijerph18010232

**Published:** 2020-12-30

**Authors:** Kavita Batra, Patricia Cruz, Chad L. Cross, Neeraj Bhandari, Farooq Abdulla, Jennifer R. Pharr, Mark P. Buttner

**Affiliations:** 1 Office of Research, School of Medicine, University of Nevada, Las Vegas, NV 89102, USA; 2 Department of Environmental and Occupational Health, University of Nevada, Las Vegas, NV 89119, USA; patricia.cruz@unlv.edu (P.C.); chad.cross@unlv.edu (C.L.C.); jennifer.pharr@unlv.edu (J.R.P.); mark.buttner@unlv.edu (M.P.B.); 3 Department of Healthcare Administration and Policy, School of Public Health, University of Nevada, Las Vegas, NV 89119, USA; neeraj.bhandari@unlv.edu; 4 Department of Pediatrics, University Medical Center of Southern Nevada, Las Vegas, NV 89102, USA; drdaddy55@aol.com

**Keywords:** neonatal abstinence syndrome, neonatal opioid withdrawal syndrome, opioid use disorder, Nevada, multilevel modelling

## Abstract

Neonatal abstinence syndrome (NAS) is a postnatal withdrawal syndrome among neonates born to mothers with drug dependence disorders. NAS poses a significant public health challenge nationally, with a six-fold increase in incidence (1.2 to 6.7 per 1000 hospital births/year) from 2000–2016. Besides national data, it is critical to quantify NAS at the state-level to identify target areas for prevention. The objectives of this study were to ascertain statewide burden, including county and regional distribution of NAS in Nevada during 2016–2018, and to investigate potential factors associated with NAS. This study utilized hospital administrative data, and a total of 100,845 inpatient pediatric discharges were examined to identify NAS cases. Statistical analyses included estimation of crude incidence rates per 1000 hospital births and multilevel logistic regression modeling. NAS incidence in Nevada decreased slightly from 8.6 to 7.7 per 1000 hospital births between 2016 and 2018, but the overall incidence of 8 was substantially higher than earlier estimates (4.8/1000 hospital births) reported for 2013. Incidence was disproportionately higher among white newborns (12, 95% CI 11.0,13.0) and Medicaid enrollees (13.2, 95% CI 11.0,15.0). Southern Nevada had the highest incidence rate of 8.2 per 1000 hospital births. Nearly 75% of NAS cases were residents of Clark County. Incidence rates of NAS parallel the growing opioid prescription rates in Nevada and highlight the need for adopting opioid control prescribing practices to combat this drug epidemic. These findings might help in designing and evaluating state- and system-level interventions introduced to combat the opioid epidemic.

## 1. Introduction

Neonatal abstinence syndrome (NAS) or neonatal opioid withdrawal syndrome (NOWS) is a constellation of withdrawal symptoms, manifested immediately after birth among babies born to mothers with drug dependence, following abrupt discontinuation of in-utero exposure to the drugs, including illegal or prescription opioids [1,2]. The risk of NAS also exists for critically ill or hospitalized infants who develop physical dependence on medications used for achieving analgesia and sedation [1]. The latter is called neonatal iatrogenic withdrawal or therapeutic NAS, which occurs secondary to therapeutic exposure of the drugs used in the neonatal intensive care units (NICU) [1,2,3]. NAS and neonatal iatrogenic withdrawal present a similar spectrum of symptoms, mainly affecting the nervous and gastrointestinal systems [3,4,5]. Symptoms of NAS/NOWS include wakefulness, irritability (high-pitched cry), tremors, hypertonic muscles, diarrhea, regurgitation (poor sucking reflex), difficulty breathing, and impaired weight gain [4,5,6]. NAS might result in long-term consequences, such as cognitive deficit and behavioral problems [7]. Death might also occur in the absence of appropriate treatment [7]. NAS results in approximately 75–95% of cases, following maternal drug use, which also increased significantly over the past few decades [1,8]. Between 1998–2011, the prevalence of maternal opioid use in the U.S. increased by 127% [9]. From 2000–2009, the antepartum use of opiates increased from 1.19 to 5.63 per 1000 births/year in the United States [8]. Consequently, there were 21,732 infants reported to be born with NAS in 2012 [10].

The incidence of NAS increased approximately 6-fold (1.20 to 6.7 per 1000 hospital births/year; 450%) from 2000–2016 [8,9,10,11]. Geographic variations of NAS incidence were also reported with the East South-Central region showing an incidence of 16.2 per 1000 hospital births, compared to the West South-Central region, with an incidence rate of 2.6 per 1000 hospital births [11]. At the state level, significant variations in NOWS incidence were also observed with an incidence as low as 0.7 cases per 1000 births in Hawaii, compared to an incidence of 33.4 cases per 1000 hospital births in West Virginia [11,12]. These state-wide variations might be due to differences in opioid prescribing rates and prevalence of opioid use disorder (OUD) among pregnant women and use of illicit substances [13]. For instance, the higher incidence of NAS in West Virginia can be partly explained by its higher opioid pain relievers (OPR) prescribing rates (137.6 prescriptions per 100 persons in 2012) and elevated incidence rates (30 cases per 1000 delivery hospitalizations/year in 2014) of OUD among reproductive-aged and pregnant women [11,12,13,14]. 

Similar trends also persist in other states. For instance, in Nevada the prevalence of OUD increased from 0.6 to 4.5 from 2002 to 2014 [13]. Nevada ranks 15th in prescribing OPRs and had a consistently higher OPR prescribing rate than the national figures (94.1 prescriptions/100 persons in Nevada vs. 82.5 OPR prescriptions/100 persons, nationally) in 2012 [14]. Correspondingly, the incidence of NAS in Nevada increased from 1.1 cases per 1000 births in 2003 to 4.8 cases per 1000 births in 2013 [12]. Our study advanced this literature in two ways. First, previous national and statewide estimates of NAS were based on the International Classification of Diseases-9 (ICD-9) coding with limited ability to differentiate NAS (secondary to maternal drug abuse) and therapeutic NAS (iatrogenic withdrawal). These estimates were provided after excluding the cases of presumed iatrogenic or therapeutic exposures [8,10] (diagnostic codes 765.00–765.05, 770.7, 772.10–772.14, 777.50–777.53, 777.6, and 779.7). Effective October 2015, the ICD-9-CM was replaced by the ICD-10-CM coding system, which offers better specificity to make a clear distinction across the two kinds of NAS. Additionally, assessment of NAS in Nevada is limited due to a lack of granularity at the county and regional levels. This study provides the most recent, precise, and comprehensive incidence estimates (including countywide data), which are important to the formulation of public health plans and allocation of health resources to improve neonatal health outcomes in Nevada. Second, we know little as to how the risk of NAS varies with demography, clinical characteristics, and hospital setting. This study explores potential patient and system-level predictors associated with NAS, which could help state and local government to more precisely tailor and target their current NAS prevention efforts.

## 2. Materials and Methods

### 2.1. Study Design and Data Source 

This study was geographically defined and employed a statewide, nested (within hospitals’ cluster), multiple cross-sectional analysis using hospital admission discharge data extracted from a de-identified state administrative database, provided by the Center for Health Information Analysis for Nevada (CHIA) [15]. As the study used de-identified data, it was considered an ‘excluded’ study (Protocol ID: 1538606) as per the Institutional Review Board criteria, according to federal regulations. Data were summarized in tables and graphs, and aggregated population-based estimates were calculated if sample sizes were adequate; any sample with n < 10 was suppressed to preserve confidentiality. 

### 2.2. Sample Selection

The population included all singleton newborn hospital (inpatient) discharge records from Nevada for the years 2016–2018. Infants with ICD-10-CM code P96.1 appearing in any diagnostic field in the hospital discharge database were included. Suspected cases of NAS, without ICD-10-CM code P96.1 and with codes P0414, P0417, and P041A were also searched (as per the Council of State and Territorial Epidemiologists definition of NAS) [16]. The following records were excluded—infants with drug withdrawal following therapeutic drug use (ICD-CM-P96.2) and newborns affected by reactions and intoxications from maternal opiates and tranquilizers used during labor and delivery (P04.0). As a reference group (non-NAS/healthy), uncomplicated births were identified using the ICD-10 code Z38.00 assigned to “single liveborn infant.”

### 2.3. Statistical Analysis

A secondary analysis of hospital administrative data was conducted. The unit of analysis was the newborn discharge/admission (in-patient) record. All statistical procedures utilized the SAS software version 9.4 (SAS Institute Inc., Cary, NC, USA). The incidence rate was determined arithmetically by dividing NAS-related newborn hospitalizations by the total number of newborn hospitalizations (with conversion to number per 1000). Overall rates (with 95% confidence intervals) are presented by demographic variables, including sex, payer source, median household income, and patient location. The median household income was approximated to the patient zip code using the most recent (2019) estimates of the American Community Survey [17]. The classification criteria (according to 2019 estimates) for income quartiles (Q1–Q4) was obtained from the Health Cost and Utilization Project (HCUP) database [18]. Patient location was categorized into large central metropolitan, medium metro, small metro, and rural (including micropolitan and noncore) according to the 2013 new county-level scheme developed by the National Center for Health Statistics (NCHS) [19]. Primary payer information was categorized as public (Medicaid), private, and uninsured (including self-pay and no charge). 

For investigating potential predictors, the primary outcome of interest was NAS (dependent variable), which was re-coded into a binary variable indicating presence (NAS = 1) or absence of NAS (NAS = 0) for the multilevel logistic regression model, to identify the factors between NAS and non-NAS births. The multilevel (2-level) logistic regression analysis was conducted using the GLIMMIX procedure to account for clustering of patients within higher-level units (i.e., hospitals). Hospital-level factors, such as hospital academic status (teaching hospital vs. non-teaching hospital), hospital location (rural vs. urban), and hospital bed size (≤100, 101–299, ≥300) were examined as factors of NAS. The categorization of hospital factors was adapted from previous studies [20,21]. In the examination of potential multilevel models, three models were built to find the best fit. The first was the null model (i.e., without any patient or hospital-related variables). This incorporated only hospital-specific random effects to model between-hospital variation in terms of NAS status [20]. The intraclass correlation coefficient (ICC) from the first model quantified the hospital-clustering effect [20,22,23]. The second model included all patient-related (level 1) variables as fixed effects, and only included the hospital-specific random intercept to examine the relationship between these variables and the incidence of NAS. A correlation matrix for all independent (level 1) variables included in the second model was calculated and examined for potential multicollinearity [24]. Upon detection of significant correlation between two variables, only one variable was included in the model. The third model included both patient and hospital factors in addition to hospital-specific random effects, and risk estimates were presented by the odds ratio [22,23].

## 3. Results

The study population consisted of 100,845 newborns discharged from 18 Nevada hospitals from 2016 to 2018. The number of newborns treated per hospital ranged from 224 to 15,542, with a median of 3762 (25th–75th percentile: 1466–9121). 

### 3.1. Incidence 

During the study period (2016–2018), NAS occurred among 796 (0.8%) inpatient pediatric discharges in Nevada. In terms of yearly variations, incidence of NAS decreased slightly from 8.6 (95% CI 8.0,9.0) to 7.7 per 1000 hospital births (95% CI 7.0,9.0) between 2016 and 2018, but the overall incidence (8.0; 95% CI 7.0,9.0) was substantially higher (1.7 times) than earlier estimates (4.8/1000 hospital births) reported for 2013. The overall NAS incidence varied across different regions, with Southern Nevada showing the highest incidence rate of 8.2 (95% CI 8.0,9.0) per 1000 hospital births, compared to other regions (northern, Washoe, and rural). In 2016, 78% of NAS cases diagnosed in Nevada were residents of Clark County (230 NAS cases in Clark vs. 292 in Nevada). From 2016 to 2018, the incidence rates declined slightly in Clark County, whereas Washoe County showed increasing trends. In 2016, the incidence of NAS in Henderson was the highest among other Nevada cities and then decreased by nearly 33% in the following two years. In the most recent study year (2018), Henderson still had the highest incidence rate among Nevada cities (i.e., 9.7 per 1000 hospital births) followed by Reno, North Las Vegas, and Las Vegas (Table 1).

Among different demographic groups, the overall incidence of NAS was the highest among white newborns, occurring at a rate of 12.0 per 1000 hospital births (95% CI 11.0, 13.0). The incidence of NAS among white infants decreased after 2016; however, a trend reversal was observed among Asian Pacific Islander (API) infants. The incidence of NAS increased from 0.9 to 3.8 per 1000 newborn hospitalizations among API infants, from 2016 to 2018. There were no differences in the overall incidence rates by gender. NAS rates also varied by income zip quartile, with infants born in the areas of the lowest quartile with a median household income of ≤$47,699 showing the highest overall incidence of NAS of 12.8 (95% CI 8.0,13.0) per 1000 hospital births. In terms of urbanization of level of residence, the NAS incidence rate was highest in large central metropolitan areas and lower but comparable among rural and small/medium metropolitan areas. Rates differed by expected payer source, with Medicaid-insured births showing the highest NAS incidence of 13.2 (95% CI 11.0,15.0) per 1000 hospital births, and 77.4% (616 out of 796) of NAS births financed by Nevada Medicaid (Table 2).

### 3.2. Predictors

In the model building process, model 3 (with level-1 & level-2 factors) appeared to be the best fit, given the progressively decreasing values of Akaike and Bayesian information criteria [24], as progression occurred from model 1 through model 3. In the unconditional model, a non-zero value of intraclass coefficient (6.5%) indicated the presence of a clustering effect of hospitals. In other words, the variability in NAS incidence attributed to the hospitals [20,24] was minimal, leaving 93.5% of the variability to be accounted for by patients (Table 3). In model 3, seven of the 13 patient characteristics (i.e., race, payer source, feeding difficulty, neonatal jaundice, seizures, transient tachypnoea, and sepsis) and none of the hospital characteristics were associated with the odds of being diagnosed with NAS. The remaining five patient level factors (i.e., gender, low birth weight, meconium aspiration syndrome, respiratory problems, and respiratory distress syndrome) were not statistically significant. In terms of race, white infants were nearly 6 times more likely to have NAS compared to black infants (OR 6.16 vs. 1.64). When examining the payer source, Medicaid insured infants were 2.8 times (OR 2.88; 95% CI 1.2–4.2) more likely to have NAS compared to those uninsured. Examination of comorbidities revealed that NAS infants had higher odds of developing transient tachypnoea, seizures, neonatal jaundice, feeding difficulties, and sepsis, compared to healthy hospital births. Finally, none of the hospital factors studied, including bed size, location (rural/urban), status (teaching/non-teaching), and type (private/public) were statistically significant (Table 4).

## 4. Discussion

In this study of infants discharged from Nevada hospitals, we observed an incidence rate of 8.6 per 1000 hospital births in 2016, which was nearly 23% greater than the national rate (7.0 per 1000 hospital births) [11,12]. Prior to 2016, the incidence rate of NAS in Nevada were slightly lower than the national rate (5.7 vs. 6.5 per 1000 hospital births in 2014). However, national estimates of NAS for the subsequent years need to be validated if incidence of NAS in Nevada is consistently higher than the rest of the nation, after 2016. The increasing incidence of NAS might partially be attributed to the variations in opioid prescribing rates, for instance, Nevada had a higher opioid prescribing rate of 80.7 prescriptions per 100 persons compared to 66.5 prescriptions per 100 persons in the U.S. in 2016 [24,25,26]. Clark County had higher opioid prescribing rates compared to Nevada in 2016 (78 vs. 73 prescriptions per 100 persons), which might have contributed to the higher NAS incidence rate in Clark County (9.1/1000 vs. 8.6/1000 hospital births) [25,26,27]. This finding has strong implications for developing countywide (particularly Clark County) evidence-based interventions to promote judicious prescribing practices, thereby reducing NAS. In 2016, the Centers for Disease Control and Prevention (CDC) provided guidelines for prescribing opioids for chronic pain management, which requires clinicians to weigh the risks and benefits associated with opioid use before prescribing to patients [28]. It also recommends that clinicians manage chronic pain with nonpharmacological and nonopioid pharmacological methods, unless the expected benefits of opioid therapy outweigh the harms associated with it [28]. If opioid pain relievers are to be prescribed, then immediate release opioids should be preferred over extended release opioids [28]. These guidelines are intended to improve the communication between clinicians and patients to raise awareness about the safe and effective use of opioid analgesics. 

Our study also reports a slight decrease in NAS incidence in 2017–2018. This decrease could represent a true plateau in the number of cases or might be due to decreased opioid prescribing rates, which dropped to 73 prescriptions per 100 persons and 55.5 prescriptions per 100 persons in 2017 and 2018, respectively [24,25,26]. The parallel trend between opioid prescribing rates and NAS is supportive of an association. These findings hint at the beneficial downstream neonatal impacts of diminished maternal opioid prescribing and underscore the continuing need of stringent regulations for preventing opioid overprescribing, such as the use of Prescription Monitoring Programs (PMPs) or by providers. Nonetheless, the potential of PMPs is not yet realized. Currently, 49 states (including Nevada) have operational PMPs; however, their utilization is low [29]. Therefore, prospective studies to assess rate of use/adoption of PMPs by providers and the effectiveness of states’ PMPs on opioid overprescribing rates can be a crucial step in controlling the opioid epidemic and neonatal sequelae, such as NAS. As expected, a higher NAS incidence was observed among Nevada Medicaid beneficiaries, which is consistent with previous reports [8,12,29,30]. This might be due to a higher prevalence of prescription opioid use among Medicaid-enrolled women of reproductive age, as opposed to those privately insured (39.4% vs. 27.7%) [31]. 

Substantial variations in NAS incidence by race also exist, with the greatest incidence rates among white infants (12.0 per 1000 births) compared to black infants (5.4 per 1000 births). These racial differences in NAS incidence might be due to higher prescription drug use among white women [30,31,32]. According to a recent Nevada-based study, the rate of emergency room visits secondary to opioid, heroin, and cannabis increased among the white population compared to other racial groups in 2016, which can help explain the racial disparity seen in terms of NAS incidence [27]. The increasing use of opioids pain relievers and other addictive substances among pregnant women warrants the need for screening and education of expectant mothers, increased access to follow-up facilities, early intervention services for high-risk mothers and infants, and for drug abuse treatment and prevention programs aimed at improving the health outcomes of mother–infant dyads.

This study also found higher incidence rates of NAS in urban counties, as compared to rural counties. These findings were not consistent with other nationwide studies [33,34]. Empirical evidence to explain this discordant finding is lacking; however, we believe that the shortage of primary care physicians, and mid-level practitioners due to a limited economic base [35,36] in rural Nevada might have contributed to the reduction in providers’ prescribed opioids and thus NAS. Additionally, the association of urbanization and maternal substance abuse is driven by multiple complex interrelationships based on macrolevel (availability of drugs and economic instability), microlevel (genetic vulnerability and personality traits), and local (family) dynamics, which cannot be uncovered by a single analysis [35,36,37]. 

### 4.1. Limitations

Our study has limitations that merit discussion. Primarily, the results of the study are not generalizable to the entire U.S., and the findings are not generalizable to deliveries occurring outside of hospitals. We suspect our county rates to be slightly underestimated because individuals living in border counties might seek medical care from neighboring states. In addition, a misclassification bias due to coding errors might have been introduced because of the use of hospital administrative data for reporting conditions or diseases in the form of billing codes. Moreover, it might be subject to underreporting because administrative data typically report fewer cases than clinical reporting. Further, the administration claimed that data lacked details to clinically assess NAS cases, in terms of severity, treatment outcomes, and type of drug exposure. Lastly, due to the unavailability of a “linkage key,” linking maternal records and neonatal records was not possible, which restricted our ability to examine maternal risk factors.

### 4.2. Strengths

To our knowledge, this was the first study to examine the distribution of NAS in Nevada, including county and city estimates. This report provides recent incidence rates of NAS, which can serve as baseline data for regional program planning and management, for reducing the burden of NAS in Nevada. Estimates provided by our study are expected to have a relatively greater precision. However, direct comparisons of our findings with estimates of prior studies should be interpreted with caution because of the transition of the ICD-9 to ICD-10 coding system, after 2015.

## 5. Conclusions

Given the continued rise of opioid use and prescribing rates among pregnant women in Nevada, the incidence rates of NAS nearly doubled in 2016–2018, as compared to previous rates reported in 2013. However, a marginal downtrend of NAS incidence was observed during the study period (2016–2018). Findings from this study have several important implications for drug abuse treatment and prevention programs aimed at improving the health outcomes of mother–infant dyads. A multifaceted approach including national-, state-, and provider-level efforts is required to curb the NAS epidemic. Provider education programs to emphasize responsible prescribing by limiting the number of days, dosage levels, prescribing alternative non-opioid pain relievers, and limiting access to refills can be beneficial in reducing the neonatal impact of prenatal opioid use. More importantly, mandated clinical reporting of Neonatal Abstinence Syndrome in Nevada is important to direct rapid preventive efforts without the time-lag associated with reporting in health insurance claim data. Clinical reporting would help document detailed maternal exposure history and the type of addictive drug used in designing targeted interventions. This study highlights the need for additional research examining the health and financial burden associated with readmission rates and long-term complications of NAS to obtain a holistic view of the problem and establish a continuum of care. It also emphasizes the need for additional regional studies to explore the multidimensional spectrum in the rural–urban context. Prospective studies to assess rate of use/adoption of PMPs by providers and effectiveness of PMPs on opioid overprescribing rates can be a crucial step in controlling the opioid epidemic and neonatal sequelae, such as NAS. 

## Figures and Tables

**Table 1:** NAS rates per 1000 births by different Nevada geographical units, 2016–2018.

Geographical Unit	Number of NAS Cases			Rate Per 1000 Hospital Births ^a^			Overall Rate Per 1000
				(95% Confidence Interval ^e^)			
	2016	2017	2018	2016	2017	2018	(2016–2018)
Statewide (Nevada)	292	247	257	8.6 (8.0, 10.0)	7.5 (7.0, 8.0)	7.7 (7.0, 9.0)	8.0 (7.0, 9.0)
Region ^b^ name							
Southern Nevada	234	194	192	9.1 (8.0, 10.0)	7.8 (7.0, 9.0)	7.7 (7.0, 9.0)	8.2 (8.0, 9.0)
Northern Nevada	12	12	10	7.1 (5.0, 17.0)	7.1 (3.0, 11.0)	7.2 (4.0, 12.0)	7.1 (5.0, 9.0)
Washoe	34	33	39	6.5 (4.0, 9.0)	6.6 (4.0, 9.0)	7.5 (5.0, 10.0)	6.8 (6.0, 8.0)
Rural Nevada	NR ^c^	NR	NR	4.1 (0, 9.0)	2.4 (0, 6.0)	5.3 (0.0, 10.0)	4.0 (1.0, 6.0)
County name							
Clark	230	191	190	9.1 (8.0, 10.0)	7.8 (7.0, 9.0)	7.7 (7.0, 9.0)	8.2 (8.0, 9.0)
Washoe	34	33	39	6.5 (4.0, 9.0)	6.5 (4.0, 9.0)	7.5 (5.0, 10.0)	6.8 (6.0, 8.0)
Others ^d^	20	18	23	7.0 (4.0, 10.0)	6.0 (3.0, 9.0)	7.9 (5.0, 11.0)	7.0 (5.0, 9.0)
City name							
Las Vegas	164	140	137	8.6 (7.0, 10.0)	7.6 (6.0, 9.0)	7.4 (6.0, 9.0)	8.0 (7.0, 9.0)
Henderson	43	29	27	15.0 (11.0, 20.0)	10.0 (6.0, 14.0)	9.7 (6.0, 13.0)	11.6 (9.0, 14.0)
North Las Vegas	18	18	23	5.6 (3.0, 8.0)	6.0 (3.0, 9.0)	7.5 (4.0, 11.0)	6.4 (5.0, 8.0)
Reno	26	21	27	7.6 (5.0, 11.0)	6.4 (4.0, 9.0)	8.0 (5.0, 11.0)	7.3 (6.0, 9.0)
Sparks	NR	NR	NR	5.0 (1.0, 9.0)	6.9 (2.0, 11.0)	5.0 (1.0, 9.0)	5.5 (3.0, 8.0)

^a^—The rates do not include suspect cases (n < 10). The suspect case is a neonate without a billing diagnosis of ICD-10 code of P96.1 diagnosis AND contains any diagnosis code of P0414, P0417, and P041A, indicating maternal use of opiates, sedative-hypnotics or anxiolytics within the birth hospitalization or a hospitalization before 28 days of age; ^b^—Southern Nevada: Clark, Esmeralda, and Nye counties; Northern Nevada: Carson City, Churchill, Douglas, Lyon, Mineral, and Storey counties; Washoe region: Washoe county; Rural Nevada Region: Elko, Eureka, Humboldt, Lincoln, Pershing, and White Pine counties; ^c^—NR = Not reported due to low volume of NAS cases (n < 10); ^d^—Other counties: Carson City, Churchill, Douglas, Elko, Esmeralda, Humboldt, Lincoln, Lyon, Mineral, Nye, Pershing, Storey, and White Pine; ^e^—lower confidence limits were truncated to “0” in the event the transformed value was negative.

**Table 2:** NAS rates per 1000 births by demographic, payer groups, and median income (2016–2018).

Group Criteria	Number of NAS Cases			Rate Per 1000 Hospital Births			Overall Rate Per 1000
				(95% Confidence Interval ^g^)			
	2016	2017	2018	2016	2017	2018	(2016–2018)
**Gender**							
Male	141	133	133	8.2 (7.0, 10.0)	7.8 (7.0, 9.0)	7.8 (7.0, 9.0)	8.0 (7.0, 9.0)
Female	149	114	124	8.9 (8.0, 10.0)	7.0 (6.0, 8.0)	7.6 (6.0, 9.0)	8.0 (7.0, 9.0)
Race							
White	217	161	172	14.0 (12.0, 16.0)	11.0 (9.0, 13.0)	11.0 (9.0, 13.0)	12.0 (11.0, 13.0)
Black	30	29	25	7.0 (5.0, 9.0)	6.5 (4.0, 9.0)	5.4 (3.0, 7.0)	6.2 (5.0, 8.0)
Hispanic	21	25	24	2.5 (1.0, 4.0)	3.3 (2.0, 5.0)	3.8 (2.0, 5.0)	3.1 (2.0, 5.0)
Asian Pacific Islander	NR ^a^	NR	NR	0.9 (0, 2.0)	3.0 (1.0, 5.0)	3.8 (1.0, 6.0)	2.5 (1.0, 4.0)
Urbanization level of residence							
Large central metro ^b^	230	191	190	9.1 (8.0, 10.0)	7.8 (7.0, 9.0)	7.7 (7.0, 9.0)	8.2 (8.0, 9.0)
Medium metro ^c^	34	33	39	6.5 (4.0, 9.0)	6.5 (4.0, 9.0)	7.5 (5.0, 10.0)	6.8 (4.0, 9.0)
Small metro ^d^	NR	NR	NR	5.5 (0, 10.0)	5.0 (0, 10.0)	3.7 (0, 9.0)	6.3 (2.0, 10.0)
Rural ^e^	16	12	16	7.9 (4.0, 12.0)	5.5 (2.0, 9.0)	7.6 (4.0, 11.0)	6.9 (5.0, 9.0)
Payer source							
Nevada Medicaid	229	197	190	14.0 (12.0, 16.0)	13.0 (11.0, 15.0)	12.7 (11.0, 14.0)	13.2 (11.0, 15.0)
Private	48	39	51	3.2 (2.0, 4.0)	2.6 (2.0, 3.0)	3.3 (2.0, 4.0)	3.1 (2.0, 4.0)
Uninsured ^f^	15	11	16	6.1 (3.0, 9.0)	3.7 (2.0, 6.0)	5.4 (2.0, 6.0)	5.0 (2.0, 6.0)
Median household income							
Quartile 1 (≤$47,999)	34	25	21	15.9 (11.0, 21.0)	12.0 (7.0, 17.0)	10.2 (6.0, 15.0)	12.8 (8.0, 13.0)
Quartile 2 ($48,000–$60,999)	136	128	125	8.4 (7.0, 10.0)	8.2 (7.0, 10.0)	7.9 (7.0, 9.0)	8.2 (7.0, 9.0)
Quartile 3 ($61,000–$81,999)	106	78	91	8.5 (7.0, 10.0)	6.2 (5.0, 8.0)	7.2 (6.0, 9.0)	7.3 (6, 8)
Quartile 4 (≥$82,000)	NR	NR	NR	5.9 (1.0, 11.0)	6.0 (1.0, 11.0)	4.9 (1.0, 9.0)	5.7 (3.0, 8.0)

^a^—Not reported due to low volume of NAS cases (n < 10); ^b^—Large central metro: Clark; ^c^—Medium Metro: Washoe & Storey; ^d^—Small metro: Carson City; ^e^—Rural: The two categories of micropolitan (Churchill, Douglas, Elko, Eureka, Humboldt, Lyon, Nye) and noncore (Esmeralda, Lander, Lincoln, Mineral, Pershing, White Pine) were combined into a single rural category to preserve the results when the sample sizes were small.; ^f^—Uninsured payer category included self-pay and no charge; ^g^—lower confidence limits were truncated to “0” in the event the transformed value was negative.

**Table 3:** Multilevel model building process.

Model	Model 1	Model 2	Model 3
Model building process			
Method	No predictors, just random effect for the intercept	Model 1 + level-1 fixed effects ^a^	Model 2 + level-2 predictors ^b^
Model fit statistics			
AIC ^c^	9179.62	4860.87	4792
BIC ^d^	9181.29	4880.87	4813
Hospital clustering statistics			
ICC ^e^	0.065 (6.5%)		

^a^—Level-1 (patient) factors: Gender, race, low birth weight, neonatal jaundice, transient tachypnoea, seizures, respiratory distress problems, respiratory difficulties, feeding difficulties, sepsis, and meconium aspiration syndrome (MAS); ^b^—Level-2 (hospital) factors: Hospital academic status, hospital location (rural vs. urban), and hospital bed size (≤100, 101–299, ≥300); ^c^—Akaike’s information criterion (AIC) used to examine model fitness. The progressive decreasing values (from model 1 to model 3) of AIC indicate improvement in model fitness; ^d^—Bayesian information criterion (BIC) used to examine model fitness. The progressive decreasing values of BIC (from model 1 to model 3) indicate improvement in model fitness; ^e^—Intraclass coefficient (ICC) was calculated manually. Formula of ICC—random intercept variance/random intercept variance + 3.29 (3.29 is the standard logistic distribution); random intercept variance 0.2295 (model 1).

**Table 4:** Estimated odds ratio for multilevel logistic regression models.

Variable	Odds Ratio ^a^ (95% Confidence Interval)	
	Model 2	Model 3
Patient characteristics		
Female	Reference	Reference
Male	0.97 (0.8–1.1)	0.97 (0.8–1.2)
Race		
Hispanic	Reference	Reference
White	6.69 (5.1–8.7)	6.16 (4.7–8.1)
Black	1.73 (1.2–2.4)	1.64 (1.2–2.3)
Asian or Pacific islander	1.32 (0.8–2.2)	1.22 (0.71–2.11)
Payer		
Self-pay/uninsured	Reference	Reference
Private	0.56 (0.4–0.8)	0.54 (0.4–0.8)
Nevada Medicaid	2.87 (2.0–4.2)	2.88 (2.0–4.2)
Comorbidities		
Feeding difficulty	4.60 (3.7–5.7)	4.54 (3.6–5.7)
Neonatal Jaundice	3.30 (2.8–4.0)	3.32 (2.8–4.0)
Seizures	2.90 (1.3–6.7)	2.96 (1.3–6.9)
Transient Tachypnoea	2.50 (2.0–3.1)	2.43 (1.9–3.1)
RDS ^b^	1.12 (0.9–1.4)	1.11 (0.9–1.4)
Sepsis	1.65 (1.2–2.3)	1.67 (1.2–2.3)
Meconium Aspiration Syndrome	1.00 (0.3–2.4)	0.98 (0.3–2.4)
Respiratory problems	0.53 (0.2–1.3)	0.53 (0.2–1.3)
Low birth weight	0.97 (0.7–1.4)	0.97 (0.7–1.4)
Hospital factors		
Bed size	-	-
≥300	-	Reference
101–299	-	0.92 (0.5–1.7)
≤100	-	0.98 (1.0–2.1)
Location		
Rural	-	Reference
Urban	-	1.37 (0.5–3.0)
Status	-	-
Non-teaching	-	Reference
Teaching	-	0.93 (0.4–1.2)
Type	-	-
Non-private	-	Reference
Private	-	0.56 (0.6–1.1)

^a^—Odds ratios are conditional or cluster-specific measures of association or intra-cluster measures of association [19]; ^b^—Respiratory distress syndrome. Note: Bold font in the Model columns indicates association.

## Data Availability

Data are publicly available.
